# Supplementary material for: NIR Instruments and Prediction Methods for Rapid Access to Grain Protein Content in Multiple Cereals
Source: Sensors (Basel). 2022 May 13;22(10):3710. doi: 10.3390/s22103710 (PMC9146900; doi:10.3390/s22103710)
Supplement: Supplementary file 1 [file sensors-22-03710-s001.zip › sensors-1632570-supplementary.pdf]

Supplementary Table S1: List of 328 grain samples used in the study, along with the crop species, genotype, and protein content (%, (g·100 g^{-1})), obtained from laboratory analysis.

S. no	Crop species	Genotype	Protein (%, (g·100 g^{-1}))
1	Finger millet	IE 2043	7.21
2	Finger millet	IE 2296	7.65
3	Finger millet	IE 2572	7.37
4	Finger millet	IE 2606	9.59
5	Finger millet	IE 2790	6.76
6	Finger millet	IE 3077	6.76
7	Finger millet	IE 3470	7.61
8	Finger millet	IE 3475	7.54
9	Finger millet	IE 3614	5.99
10	Finger millet	IE 3618	7.58
11	Finger millet	IE 4057	8.01
12	Finger millet	IE 4073	7.39
13	Finger millet	IE 4115	7.55
14	Finger millet	IE 4121	8.78
15	Finger millet	IE 4671	9.07
16	Finger millet	IE 5066	9.50
17	Finger millet	IE 5106	8.88
18	Finger millet	IE 5165	8.65
19	Finger millet	IE 518	7.62
20	Finger millet	IE 5367	9.00
21	Foxtail millet	ISe 1251	10.96
22	Foxtail millet	ISe 1454	11.08
23	Foxtail millet	ISe 1468	9.08
24	Foxtail millet	ISe 1511	11.13
25	Foxtail millet	ISe 1664	12.39
26	Foxtail millet	ISe 1805	12.45
27	Foxtail millet	ISe 1881	12.66
28	Foxtail millet	ISe 1892	12.91
29	Foxtail millet	ISe 238	11.92
30	Foxtail millet	ISe 289	10.39
31	Foxtail millet	ISe 480	10.86
32	Foxtail millet	ISe 525	10.52
33	Foxtail millet	ISe 719	11.89
34	Foxtail millet	ISe 783	13.42
35	Foxtail millet	ISe 796	11.65
36	Foxtail millet	ISe 827	13.17
37	Foxtail millet	ISe 828	11.40
38	Foxtail millet	ISe 840	9.89
39	Foxtail millet	ISe 869	10.82
40	Maize	783527	9.34
41	Maize	4695575	9.97
42	Maize	9424780	9.62

43	Maize	18270413	9.04
44	Maize	22525674	9.10
45	Maize	900MG	8.98
46	Maize	X35D602	8.76
47	Maize	X35D612	8.53
48	Maize	X35D620	8.81
49	Maize	X35F833	9.21
50	Pearl millet	9444	12.53
51	Pearl millet	PUSA 322	9.69
52	Pearl millet	86 M 86	14.41
53	Pearl millet	86 M 88	11.42
54	Pearl millet	863B	11.65
55	Pearl millet	863B-P2	15.19
56	Pearl millet	APH 45	10.45
57	Pearl millet	Bio 451	10.29
58	Pearl millet	Bio 549	10.61
59	Pearl millet	BLMPH 105	10.16
60	Pearl millet	GB8735	10.87
61	Pearl millet	ICMV 93191	16.12
62	Pearl millet	GK 1183	11.75
63	Pearl millet	GK 1207	10.41
64	Pearl millet	GK 1235	11.02
65	Pearl millet	H77/833-2	9.76
66	Pearl millet	HT 416628	12.31
67	Pearl millet	HYMH 5	12.67
68	Pearl millet	HYMH 8	12.24
69	Pearl millet	ICMB 89111-P6	15.00
70	Pearl millet	ICMB 90111-P2	15.54
71	Pearl millet	ICMB 90111-P6	13.45
72	Pearl millet	ICML 22	14.72
73	Pearl millet	ICMP 451-P6	15.72
74	Pearl millet	ICMP 451-P8	14.97
75	Pearl millet	ICMS 7703	21.51
76	Pearl millet	ICMS 7704	19.84
77	Pearl millet	ICMV 155	15.05
78	Pearl millet	ICMV 221	19.04
79	Pearl millet	ICMV-IS 92222	21.16
80	Pearl millet	IP 10085	18.74
81	Pearl millet	IP 10394	15.42
82	Pearl millet	IP 10446	16.11
83	Pearl millet	IP 10539	15.33
84	Pearl millet	IP 10705	16.08
85	Pearl millet	IP 10761	14.73
86	Pearl millet	IP 10811	15.94
87	Pearl millet	IP 10953	13.54
88	Pearl millet	IP 11211	17.96

89	Pearl millet	IP 11275	16.95
90	Pearl millet	IP 11577	19.20
91	Pearl millet	IP 12116	15.82
92	Pearl millet	IP 12322	19.77
93	Pearl millet	IP 12840	18.46
94	Pearl millet	IP 13384	16.13
95	Pearl millet	IP 13964	18.86
96	Pearl millet	IP 15551	16.91
97	Pearl millet	IP 15946	18.04
98	Pearl millet	IP 16082	16.09
99	Pearl millet	IP 16096	17.03
100	Pearl millet	IP 16403	18.14
101	Pearl millet	IP 17611	16.02
102	Pearl millet	IP 17632	13.49
103	Pearl millet	IP 17720	18.00
104	Pearl millet	IP 18062	17.19
105	Pearl millet	IP 18132	16.65
106	Pearl millet	IP 18168	17.32
107	Pearl millet	IP 18293-P152	18.50
108	Pearl millet	IP 19386	15.97
109	Pearl millet	IP 19388	16.25
110	Pearl millet	IP 19405	14.59
111	Pearl millet	IP 19448	16.89
112	Pearl millet	IP 21517	15.34
113	Pearl millet	IP 22423	14.79
114	Pearl millet	IP 22424	14.57
115	Pearl millet	IP 22455	17.43
116	Pearl millet	IP 3108	18.05
117	Pearl millet	IP 3125	14.12
118	Pearl millet	IP 3175	17.14
119	Pearl millet	IP 3509	17.44
120	Pearl millet	IP 3616	16.10
121	Pearl millet	IP 3732	17.46
122	Pearl millet	IP 4020	21.46
123	Pearl millet	IP 4927	18.72
124	Pearl millet	IP 4979	14.92
125	Pearl millet	IP 5207	19.00
126	Pearl millet	IP 5253	12.76
127	Pearl millet	IP 5713	16.18
128	Pearl millet	IP 5923	17.39
129	Pearl millet	IP 6060	17.51
130	Pearl millet	IP 6102	18.34
131	Pearl millet	IP 6110	19.48
132	Pearl millet	IP 6112	16.76
133	Pearl millet	IP 6146	18.34
134	Pearl millet	IP 6179	17.86

135	Pearl millet	IP 6310	16.99
136	Pearl millet	IP 6460	18.65
137	Pearl millet	IP 6682	16.39
138	Pearl millet	IP 6769	16.41
139	Pearl millet	IP 6891	20.14
140	Pearl millet	IP 7470	16.50
141	Pearl millet	IP 7633	16.91
142	Pearl millet	IP 7762	16.45
143	Pearl millet	IP 7941	16.51
144	Pearl millet	IP 7970	15.79
145	Pearl millet	IP 8129	17.50
146	Pearl millet	IP 8166	14.77
147	Pearl millet	IP 8198	16.09
148	Pearl millet	IP 8210	15.88
149	Pearl millet	IP 8276	15.37
150	Pearl millet	IP 8426	17.13
151	Pearl millet	IP 8761	19.07
152	Pearl millet	IP 8767	17.37
153	Pearl millet	IP 8786	19.55
154	Pearl millet	IP 8972	16.53
155	Pearl millet	IP 9282	17.34
156	Pearl millet	IP 9347	17.27
157	Pearl millet	IP 9407	19.43
158	Pearl millet	IP 9426	20.31
159	Pearl millet	IP 9446	15.57
160	Pearl millet	IP 9651	15.56
161	Pearl millet	IP 9692	17.98
162	Pearl millet	IP 9710	15.67
163	Pearl millet	IP 9840	16.63
164	Pearl millet	IP 9854	15.93
165	Pearl millet	J 104	19.42
166	Pearl millet	JKBH 1352	9.72
167	Pearl millet	JKBH 1490	13.44
168	Pearl millet	KH 3022	11.19
169	Pearl millet	NBH 5863	10.48
170	Pearl millet	NU 399	10.62
171	Pearl millet	NU 409	10.65
172	Pearl millet	PRLT	10.09
173	Pearl millet	Super Boss	11.91
174	Pearl millet	Tift 383	15.97
175	Sorghum	Keninkeni	10.62
176	Sorghum	00-CZ-F5P-135	12.55
177	Sorghum	01-BE-F5P-15	12.07
178	Sorghum	02-SB-F4DT-275	13.09
179	Sorghum	296B	10.48
180	Sorghum	98-BE-F5P-84	11.67

181	Sorghum	AKSV BR (PKV KRANTI)	11.73
182	Sorghum	B2-3	9.04
183	Sorghum	B2-5	10.69
184	Sorghum	B35	11.07
185	Sorghum	BJV44	9.61
186	Sorghum	BTx623	10.62
187	Sorghum	C41-28-49-11(D3)	12.67
188	Sorghum	C41-28-49-14(D1)	11.18
189	Sorghum	C41-28-49-18-7	16.55
190	Sorghum	C41-28-49-18-9	13.10
191	Sorghum	C41-28-49-27-1	13.67
192	Sorghum	C41-28-52-13-5	11.50
193	Sorghum	C41-28-52-18(D3)	10.98
194	Sorghum	C41-28-52-26-1	12.93
195	Sorghum	C41-28-52-28-2	12.28
196	Sorghum	C41-28-52-28-3	12.21
197	Sorghum	C41-28-52-28-5	12.20
198	Sorghum	C41-28-75-21(D1)	13.32
199	Sorghum	C41-28-75-26-4	12.08
200	Sorghum	CIRAD406	12.00
201	Sorghum	CMDT45	11.42
202	Sorghum	CRS4	10.42
203	Sorghum	CSH16	11.25
204	Sorghum	CSM388	14.46
205	Sorghum	CSM63-E	11.46
206	Sorghum	CSV 14R	11.91
207	Sorghum	CSV 18	13.42
208	Sorghum	CSV 216R	9.23
209	Sorghum	CSV 26	13.01
210	Sorghum	CSV22	13.68
211	Sorghum	CSV29R	13.24
212	Sorghum	Doua-G	8.79
213	Sorghum	E36-1	10.09
214	Sorghum	E36-1	12.04
215	Sorghum	Framida	12.64
216	Sorghum	Gnossiconi	10.74
217	Sorghum	GPN01 S01 266-2-1-6-vr	8.68
218	Sorghum	GPN01 S01 267-9-3-1-1	10.51
219	Sorghum	GPN01 S01 267-9-3-3-vr	9.71
220	Sorghum	GRS1 (DSV5)	10.58
221	Sorghum	GS15-10	11.21
222	Sorghum	GS23	10.59
223	Sorghum	ICSB 370-2-9	10.38
224	Sorghum	ICSV745	10.65
225	Sorghum	ICSV93046-P1	10.29
226	Sorghum	IS 41397-3-P6	11.02

227	Sorghum	IS 8219-P1	9.34
228	Sorghum	IS10876	12.45
229	Sorghum	IS11026	17.90
230	Sorghum	IS11473	17.01
231	Sorghum	IS11919	17.25
232	Sorghum	IS12804	14.11
233	Sorghum	IS12883	13.43
234	Sorghum	IS12965	17.06
235	Sorghum	IS13893	13.54
236	Sorghum	IS14556	11.82
237	Sorghum	IS14779	14.05
238	Sorghum	IS15401	9.65
239	Sorghum	IS15466	11.41
240	Sorghum	IS15945	14.31
241	Sorghum	IS16528	15.58
242	Sorghum	IS25249	17.78
243	Sorghum	IS25548	16.24
244	Sorghum	IS25910	17.85
245	Sorghum	IS25989	18.22
246	Sorghum	IS26046	16.54
247	Sorghum	IS26222	16.05
248	Sorghum	IS26617	15.34
249	Sorghum	IS26694	14.40
250	Sorghum	IS26701	13.69
251	Sorghum	IS26737	14.44
252	Sorghum	IS27557	17.65
253	Sorghum	IS27786	17.10
254	Sorghum	IS27887	15.04
255	Sorghum	IS27912	14.45
256	Sorghum	IS28141	14.82
257	Sorghum	IS28313	17.35
258	Sorghum	IS28389	17.01
259	Sorghum	IS28449	17.40
260	Sorghum	IS28614	18.38
261	Sorghum	IS28747	17.88
262	Sorghum	IS28849	15.85
263	Sorghum	IS29091	12.29
264	Sorghum	IS29100	14.85
265	Sorghum	IS29187	12.50
266	Sorghum	IS29304	13.86
267	Sorghum	IS29314	14.64
268	Sorghum	IS29472	11.47
269	Sorghum	IS29568	17.50
270	Sorghum	IS29606	14.16
271	Sorghum	IS29689	15.39
272	Sorghum	IS30231	15.04

273	Sorghum	IS30460	12.38
274	Sorghum	IS30507	15.59
275	Sorghum	IS30572	16.80
276	Sorghum	IS30838	14.32
277	Sorghum	IS31186	12.59
278	Sorghum	IS31446	15.59
279	Sorghum	IS31681	16.64
280	Sorghum	IS31706	16.06
281	Sorghum	IS32787	13.46
282	Sorghum	IS33023	15.97
283	Sorghum	IS33090	14.84
284	Sorghum	IS393(411)695	10.93
285	Sorghum	IS7957	13.86
286	Sorghum	IS8012	13.09
287	Sorghum	IS9113	14.66
288	Sorghum	*Sevata jonna* (landrace)	12.66
289	Sorghum	M35-1	9.69
290	Sorghum	M35-1	10.75
291	Sorghum	M35-1	11.55
292	Sorghum	N13	10.26
293	Sorghum	Parbahani Moti	10.54
294	Sorghum	Parbhani Jyothi	10.62
295	Sorghum	PB15220-1	10.61
296	Sorghum	PB15881-3	10.45
297	Sorghum	Phule Anuradah (RSV 458)	13.79
298	Sorghum	Phule Chitra (SPV 1546)	13.36
299	Sorghum	Phule Maulee (RSLG262)	10.48
300	Sorghum	Phule Maulee	12.82
301	Sorghum	Phule Revati (RSV1006)	13.62
302	Sorghum	Phule Vasudha	17.99
303	Sorghum	Phule Vasudha	9.97
304	Sorghum	PVK 801-P23	11.75
305	Sorghum	R16	10.87
306	Sorghum	R16	16.39
307	Sorghum	R37-13-11-2-21-2	12.12
308	Sorghum	R37-13-11-2-26(D3)	13.35
309	Sorghum	R37-13-11-2-30(D2)	14.06
310	Sorghum	R37-13-11-2-40	13.34
311	Sorghum	R37-13-11-2-40(D1)	14.25
312	Sorghum	R37-13-11-2-40(D3)	14.08
313	Sorghum	R37-13-11-2-5-1	14.34
314	Sorghum	R37-13-30-1(D6)	12.60
315	Sorghum	R37-13-30-1(D7)	12.62
316	Sorghum	R37-13-30-11-4	11.91
317	Sorghum	R37-13-30-11-6	11.93
318	Sorghum	R37-13-30-15(D3)	12.56

319	Sorghum	R37-13-30-16(D2)	11.62
320	Sorghum	R37-13-30-28(D3)	15.09
321	Sorghum	Ribdahu	10.53
322	Sorghum	RSLG262	13.59
323	Sorghum	S35	11.65
324	Sorghum	S35	15.17
325	Sorghum	Sambalma	9.83
326	Sorghum	SP 2417-P3	10.41
327	Sorghum	SPV2217	10.43
328	Sorghum	SVD806	10.32

Supplementary Table S2: Descriptive statistics presenting the variability and range of protein content in the calibration and validation sets used in the study. Legend: SD = standard deviation; CV% = coefficient of variation.

**Details**	**Training set**	**Validation set**
Number of samples	262	66
Range of protein content (%, (g·100 g^{-1}))	5.99–21.51	6.76–20.14
Average (%, (g·100 g^{-1}))	13.67	13.27
SD	3.25	3.37
CV%	23.79	25.36

Supplementary Table S3: Descriptive statistics (minimum (min), maximum (max), average (avg), standard deviation (SD), and standard error (SE)) of the protein content (%, (g·100 g^{-1})) for each of the cereal species of the calibration and validation sets used in the study.

Set	n	Protein content (%, (g·100 g^{-1}))				
		Min	Max	Avg	SD	SE
**Calibration**	**262**	**5.99**	**21.51**	**13.67**	**3.25**	**0.20**
Finger millet	16	5.99	9.59	7.95	0.94	0.24
Foxtail millet	16	9.08	13.42	11.49	1.16	0.29
Maize	7	8.76	9.97	9.23	0.44	0.17
Pearl millet	99	9.69	21.51	15.88	2.74	0.28
Sorghum	124	8.68	18.38	13.17	2.45	0.22
**Validation**	**66**	**6.76**	**20.14**	**13.27**	**3.37**	**0.41**
Finger millet	4	6.76	9.07	7.84	1.26	0.63
Foxtail millet	3	10.52	13.17	11.59	1.40	0.81
Maize	3	8.53	9.21	8.91	0.35	0.20
Pearl millet	26	9.76	20.14	15.38	3.23	0.63
Sorghum	30	8.79	17.40	12.77	2.35	0.43
**Total**	**328**	**5.99**	**21.51**	**13.59**	**3.27**	**0.18**

Supplementary Table S4: Comparative metrics of NIR spectroscopy calibration (80%) and validation (20%) models developed using combinations of two different instruments (FOSS-DS2500 and HL-EVT5) and Hone Create software for protein content estimation in grains of multiple cereal species. Legend: R^2 = coefficient of determination; RMSE = Root Mean Squared Errors, RPD = ratio of prediction to deviation; Best prediction model for each of the instruments is highlighted in bold.

Instrument	Model type	Spectrum merging step	Pre-processing step	Calibration Set					Validation Set				
				RMSE	R^2	Slope	Intercept	RPD	RMSE	R^2	Slope	Intercept	RPD
FOSS-DS2500	Stacked ensemble	-	Smoothing	1.36	0.82	0.82	2.51	2.39	1.67	0.76	0.70	3.98	2.02
			Baseline Correction	1.30	0.84	0.83	2.34	2.51	1.51	0.80	0.74	3.32	2.23
			Area Normalization	1.11	0.88	0.89	1.50	2.92	1.33	0.84	0.79	2.77	2.52
			Standard Normal Variate	0.86	0.93	0.93	0.91	3.76	1.24	0.86	0.82	2.34	2.71
			**Derivative**	**0.66**	**0.96**	**0.95**	**0.64**	**4.93**	**1.00**	**0.91**	**0.89**	**1.44**	**3.38**
HL-EVT5	Stacked ensemble	-	Derivative	0.38	0.99	0.97	0.43	8.66	1.00	0.91	0.88	1.62	3.38
		Smoothing and Derivative	Baseline Correction	0.55	0.97	0.96	0.58	5.90	0.98	0.91	0.89	1.44	3.42
			Standard Normal Variate	0.40	0.98	0.97	0.42	8.04	0.98	0.92	0.90	1.42	3.44
			Smoothing	0.42	0.98	0.97	0.43	7.79	0.97	0.92	0.90	1.35	3.48
			**Area Normalization**	**0.52**	**0.97**	**0.96**	**0.54**	**6.28**	**0.94**	**0.92**	**0.90**	**1.39**	**3.57**
